# Application of Aligned-UMAP to longitudinal biomedical studies

**DOI:** 10.1016/j.patter.2023.100741

**Published:** 2023-05-08

**Authors:** Anant Dadu, Vipul K. Satone, Rachneet Kaur, Mathew J. Koretsky, Hirotaka Iwaki, Yue A. Qi, Daniel M. Ramos, Brian Avants, Jacob Hesterman, Roger Gunn, Mark R. Cookson, Michael E. Ward, Andrew B. Singleton, Roy H. Campbell, Mike A. Nalls, Faraz Faghri

**Affiliations:** 1 Department of Computer Science, University of Illinois at Urbana-Champaign, Champaign, IL 61820, USA; 2 Center for Alzheimer’s and Related Dementias (CARD), National Institute on Aging and National Institute of Neurological Disorders and Stroke, National Institutes of Health, Bethesda, MD 20892, USA; 3 Data Tecnica International, Washington, DC 20037, USA; 4 Department of Industrial and Enterprise Systems Engineering, University of Illinois at Urbana-Champaign, Champaign, IL 61820, USA; 5 Laboratory of Neurogenetics, National Institute on Aging, National Institutes of Health, Bethesda, MD 20892, USA; 6 Invicro, Image Analysis, Needham, MA, USA; 7 National Institute of Neurological Disorders and Stroke, National Institutes of Health, Bethesda, MD, USA

**Keywords:** machine learning, unsupervised learning, longitudinal data, time-series, Parkinson's disease, Alzheimer's disease, clinical data, proteomics, genomics, iPSC

## Abstract

High-dimensional data analysis starts with projecting the data to low dimensions to visualize and understand the underlying data structure. Several methods have been developed for dimensionality reduction, but they are limited to cross-sectional datasets. The recently proposed Aligned-UMAP, an extension of the uniform manifold approximation and projection (UMAP) algorithm, can visualize high-dimensional longitudinal datasets. We demonstrated its utility for researchers to identify exciting patterns and trajectories within enormous datasets in biological sciences. We found that the algorithm parameters also play a crucial role and must be tuned carefully to utilize the algorithm’s potential fully. We also discussed key points to remember and directions for future extensions of Aligned-UMAP. Further, we made our code open source to enhance the reproducibility and applicability of our work. We believe our benchmarking study becomes more important as more and more high-dimensional longitudinal data in biomedical research become available.

## Highlights

• We explore the utility of Aligned-UMAP in longitudinal biomedical datasets
• This overview offers insights on optimal uses for the technique
• We make recommendations for best practices when using Aligned-UMAP

## The bigger picture

Longitudinal multi-dimensional biological datasets are ubiquitous and highly abundant. These datasets are essential to understanding disease progression, identifying subtypes, and discovering drugs. Discovering meaningful patterns or disease pathophysiologies in these datasets is challenging due to their high dimensionality, making it difficult to visualize hidden patterns. In this work, we applied Aligned-UMAP on a broad spectrum of clinical, imaging, proteomics, and single-cell datasets. Aligned-UMAP reveals time-dependent hidden patterns when color coded with the metadata. Altogether, based on its ease of use and our evaluation of its performance on different modalities, we anticipate that Aligned-UMAP will be a valuable tool for the biomedical community.

## Graphical abstract

**Figure:**
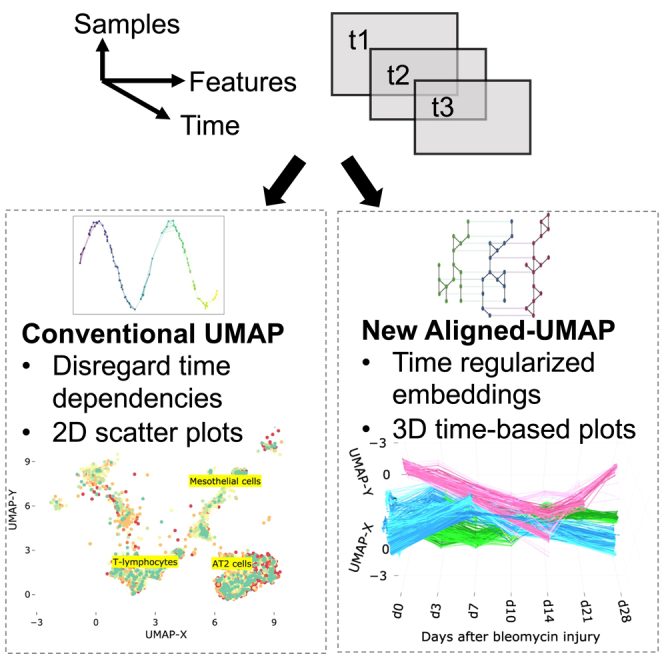

## Teaser

High-dimensional longitudinal data are prevalent yet understudied in biological literature. Discovering meaningful patterns from these datasets is an important task. Though few methods are available for visualizing high-dimensional longitudinal data, they are not studied extensively in real-world biological datasets. A recently developed nonlinear dimensionality reduction technique, Aligned-UMAP, analyzes sequential data. Here, we give an overview of applications of Aligned-UMAP on various biomedical datasets.

## Introduction

Visualizing large-scale, high-dimensional datasets is the starting step for any data exploratory analysis. Visualizing data is particularly useful for the biological community, where researchers rely on hypothesis-free data-driven analytics to gain essential insights and observe meaningful patterns from the data. The standard way of visualizing high-dimensional data is to project the data into low-dimensional space, typically 2D or 3D, while preserving local and global relationships. This transformation is called dimension reduction and belongs to the unsupervised machine learning algorithms class. The lower-dimensional data space can guide us in various tasks, such as identifying clusters, substructures, and outliers; detecting batch effects; and quality control measures to perform reliable and accurate downstream analyses.

In contrast to traditional methods for dimensionality reduction—for example, principal-component analysis (PCA)^1^—uniform manifold approximation and projection (UMAP)^2^ learns a nonlinear embedding of the original space by optimizing the embedding coordinates of individual data points using iterative algorithms. It aims to accurately preserve the original local neighborhood of each data point in the visualization. Because of the expressiveness of nonlinear embeddings, UMAP is well regarded for its state-of-the-art empirical performance at elucidating sophisticated manifold structures. The biomedical community widely adopts UMAP for multiple studies ranging from single-cell RNA sequencing (RNA-seq) data^3^ to genetics^4^^,^^5^ or complex clinical symptoms^3^^,^^6^ to depict exciting patterns from the data. In these use cases, UMAP is explored on datasets assuming that all samples in the dataset are independent.

Despite the prevalence of nonindependent high-dimensional biological datasets, the application of UMAP in this area is little explored. This nonindependence effect can occur from measurements at different time intervals, age, or other discrete/continuous variables. There are various longitudinal datasets of different modalities such as clinical symptoms, magnetic resonance imaging (MRI), electronic health records (EHRs), electroencephalography (EEG) for sleep monitoring, electrocardiogram (ECG) data, etc. Since UMAP is a stochastic algorithm, different runs with the same hyperparameters can yield different results; therefore, extension to longitudinal datasets is not straightforward, unlike traditional algorithms such as PCA. Aligned-UMAP is a recently introduced dimensionality reduction approach for temporal data by the authors of UMAP (https://umap-learn.readthedocs.io/en/latest/aligned_umap_basic_usage.html). It is based on the UMAP^2^ and MAPPER^7^ algorithms. MAPPER is a well-known topological data analysis method that successfully studies temporal, unbiased transcriptional regulation patterns.^8^ Aligned-UMAP imposes time constraints in the low-dimensional embeddings, thereby controlling the stochasticity of its cross-sectional counterpart along the longitudinal axis. TimeCluster^9^ is another approach that reduces the dimensionality of time-series data. Though it is possible to discover clusters with similar trajectories using TimeCluster, their intrinsic longitudinal variation cannot be observed. Further, it requires data availability for every time instance, making it less applicable for most biological datasets.

In this work, we deep dive into the applications of Aligned-UMAP on various longitudinal biological datasets. We applied the algorithm to clinical data, brain images, longitudinal proteomic data, EHRs, and ECG datasets. We demonstrated its utility for researchers to identify exciting patterns and trajectories within enormous datasets. Secondly, we show the effect of different parameters of Aligned-UMAP on the lower-dimension space. We also performed computation time analysis with varying datasets as a factor of the number of CPU cores. Furthermore, we deployed an interactive data visualization tool for reproducibility and transparency, motivated by open science. A deeper investigation of observed patterns could reveal more detailed, meaningful information, which is out of the scope of this work.

## Results

### Overview of the Aligned-UMAP method

#### UMAP

UMAP is a dimensionality reduction method that learns a nonlinear low-dimensional embedding of the original high-dimensional space. UMAP has solid theoretical foundations based on manifold theory and tries to preserve both local and some global structures better than other popular techniques such as t-distributed stochastic neighbor embedding (t-SNE). UMAP is a graph-based dimensionality reduction method. It has two phases—first, computation of a weighted nearest-neighbor graph from the high-dimensional dataset. In the second phase, a low-dimensional layout is computed by optimizing the objective function that preserves desired characteristics of this nearest-neighbor graph. The algorithm is computationally efficient with the time order of sample size for the low-dimensional optimization phase but is essentially bounded by the log-linear complexity of the nearest-neighbor search phase in practical scenarios.^10^ It is the superior run time performance of UMAP compared with its counterparts that makes it very popular among the dimensionality reduction methods.^3^

#### Aligned-UMAP

**Figure 1:**
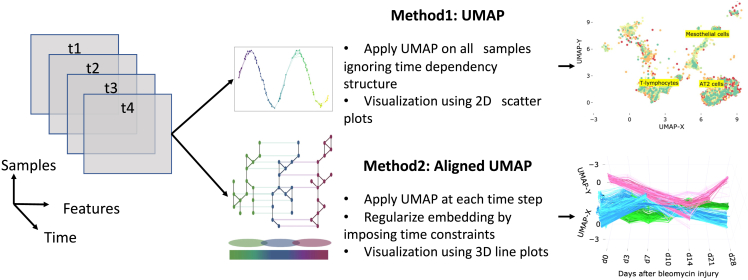
The workflow of analysis and model development

Aligned-UMAP is a recently introduced dimensionality reduction approach for temporal data. The trivial way of performing dimensionality reduction on longitudinal data is to apply UMAP independently at different time steps and align the embedding using a Procrustes transformation on related points. However, Aligned-UMAP optimizes both embeddings simultaneously using a regularizer term to provide better alignments in general. The MAPPER algorithm is used to get the regularizer term, which enforces the constraint on how far related points can take different locations in embeddings at multiple time points. Further details for the algorithm can be found on the UMAP documentation website (https://umap-learn.readthedocs.io/en/latest/aligned_umap_basic_usage.html). Figure 1 shows the pipeline of our analysis workflow.

### Software output and reproducibility

A demo of the Aligned-UMAP visualization is available at https://alignedumap-biomedicaldata.streamlit.app. The data analysis pipeline for this work was performed in Python 3.8 using open-source libraries (numpy, pandas, plotly, umap). Our code is publicly available at https://github.com/NIH-CARD/AlignedUMAP-BiomedicalData to facilitate replication and future expansion of our work. The repository is well documented and includes a description of the data preprocessing, statistical, and machine learning analyses used in this study.

### Visualizing high-dimensional longitudinal data

We study Aligned-UMAP in a wide range of biomedical datasets from multiple data modalities. Table 1 shows the statistics of various datasets, with the count of samples ranging from approximately 500 to 21,000. These datasets vary in both the number of time sequences and the number of features available. For every visualization, each representative point becomes a thread through the time axis as their relative position changes in the low-dimensional space.

**Table 1:** Dataset overview and statistics

Dataset	Modality	No. samples	No. features	No. time sequences
PPMI clinical data^11^^,^^12^^,^^13^	clinical assessment	476	122	6
ADNI clinical data^14^^,^^15^	clinical assessment	435	78	4
PPMI-ADNI T1 MRI^13^^,^^15^^,^^16^	MRI T1 imaging	2,836	406	52
MIMIC-III^17^	EHR	36,675	64	6
Longitudinal proteomic COVID-19^18^	proteomics	383	1,463	3
Longitudinal whole-lung scRNA^19^	scRNA	10,111	21,767	7
iPSC-derived neurons^20^	proteomics	18	4,959	6

#### Clinical data

In neurodegenerative diseases such as Alzheimer’s and Parkinson’s, the individual can manifest disease in various ways, oftentimes prior to clinical diagnosis. We evaluate the Aligned-UMAP algorithm on the clinical assessment data from Alzheimer’s Disease Neuroimaging Initiative (ADNI) and Parkinson’s Progression Marker Initiative (PPMI) study cohorts. The ADNI study includes patients with Alzheimer’s, mild cognitive impairment subjects, and elderly controls. The PPMI study has subjects recently diagnosed with Parkinson’s disease (PD) and healthy controls. These studies collect data for many clinical assessments related to movement and cognitive disability to monitor disease progression. All such measurements are recorded longitudinally at separate visits. The time duration of such visits can range from years to decades.

**Figure 2:**
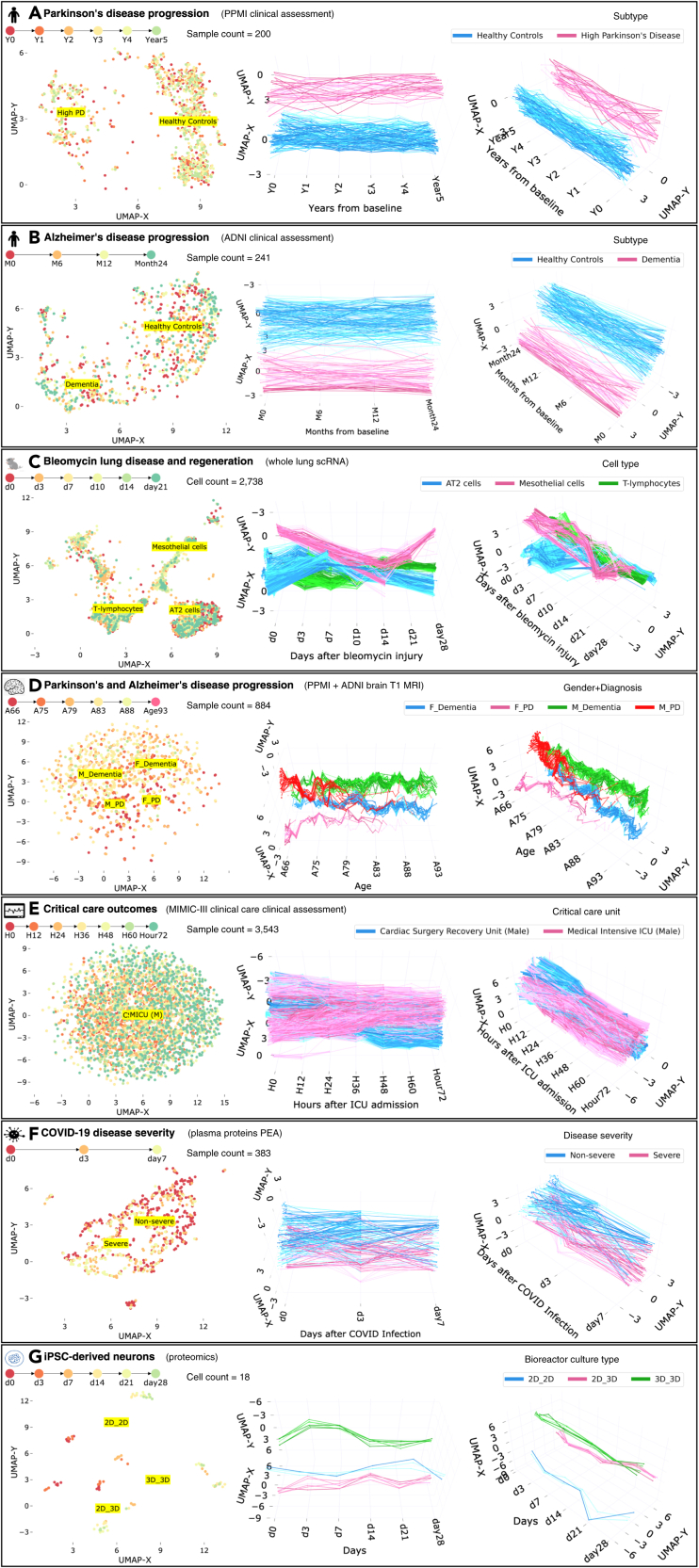
Low-dimensional embeddings by UMAP and Aligned-UMAP dimensionality reduction algorithms on longitudinal biomedical datasets from multiple modalities (A) The distinction between Parkinson’s disease subjects (with rapid progressors) and healthy controls from 122 clinical measurements collected over 5 years from Parkinson’s Progression Markers Initiative (PPMI) study. Measures include MoCA scores and MDS-Unified Parkinson’s Disease Rating Scale scores. (B) Trajectories of dementia and healthy control subjects on 78 clinical measurements collected over 2 years from the Alzheimer’s Disease Neuroimaging Initiative (ADNI) study. Measurements include Mini-Mental State Exam (MMSE) scores and Alzheimer’s Disease Assessment Scale-Cognitive Subscale (ADAS-COG) tests. (C) Aligned-UMAP trajectories show shifts in specific cell types (such as mesothelial and AT2 cells) in gene expression space during the regeneration time course of mice having bleomycin lung injury. (D) Aligned-UMAP embeddings depict aging patterns for patients with dementia and Parkinson’s disease, stratified by gender. (E) Trajectories of the subjects admitted in different critical care units of the MIMIC-III database. Measurements include vital signs such as blood pressure, oxygen levels, and ICD-9 diagnosis codes. (F) Embedding space depicts the severity of COVID-19 disease from 1,463 unique plasma proteins measured by proximity extension assay using the Olink platform. The cutoff at day 3 is visible because of data unavailability at day 7 due to either patient recovery or death. (G) Aligned-UMAP low-dimensional space identified the cell culture environment of iPSC-derived neurons using longitudinal proteomic data for more than 8,000 proteins. Note: we apply the Aligned-UMAP algorithm on the dataset having characteristics shown in Table 1. In this figure, we have demonstrated a subset of classes for better visualization purposes. For more detailed analysis, users can explore our public web application.

We preprocess the ADNI and PPMI cohort datasets following the strategy proposed in previous disease subtyping studies.^11^^,^^12^^,^^14^ UMAP and Aligned-UMAP successfully pulled together clusters corresponding to populations with similar disease progression (Figures 2A and 2B). However, longitudinal differences got lost in the UMAP version due to its stochastic nature. Aligned-UMAP separates the rapidly progressive PD subgroup from the healthy control group and demonstrates divergence of the rapid PD subgroup from healthy controls with aging (Figure 2A). Furthermore, Aligned-UMAP reveals distinct longitudinal courses for dementia and the healthy control group (Figure 2B). We follow a continuum spectrum from lower progressive to high progressive subgroups for PD and dementia subjects. These results suggest that Aligned-UMAP could be used as a hypothesis-generating tool to identify distinct subtypes based on disease progression. For instance, a particular subgroup shows rapid decline in clinical symptoms such as MDS-Unified Parkinson’s Disease Rating Scale^21^ or MoCA cognitive assessment^22^ compared with healthy control and other subgroups.

#### Whole-lung single-cell RNA (scRNA) data

Single-cell transcriptomics (scRNA) using next-generation transcript sequencing (RNA-seq) has recently received much attention due to its ability to uncover cellular heterogeneity, cellular differentiation, and development mechanisms. UMAP has demonstrated its efficacy in analyzing single-cell datasets by identifying clusters of related cells. Modeling gene expression trajectories of different cell types have been successfully used to understand cell-cell communication routes in various chronic diseases such as lung disease and tumor cells.^19^^,^^23^ We evaluated Aligned-UMAP on whole-lung scRNA data of mice undergoing regeneration after bleomycin-induced lung injury.^19^ Transcriptomic profiles of 29,297 cells were collected from six time points (days 3, 7, 10, 14, 21, and 28). We observe clusters of cell types showing different cellular dynamics through the regeneration process (Figure 2C); mesothelial cells show a spike at day 14 and start returning to their healthy state (day 0), thereby suggesting the role of mesothelial cells in bleomycin-related lung injury. This way, we could extract hidden longitudinal patterns from high-dimensional time-series datasets using Aligned-UMAP.

#### Imaging data

Imaging is a pervasive way of monitoring the disease progression of multiple disorders. We use the advanced normalization tools (ANTs) pipeline^16^ to extract structural features such as the volume and area of different brain regions from the MRI T1 image. Since the number of longitudinal images for each subject is scarce, we use the imaging features to model aging trajectories. To be precise, we relate images if they are observed at similar age groups instead of relating subjects based on their visits. Also, these relations are constrained by different diagnosis groups (i.e., control, PD, or dementia). Figure 2D shows various aging courses based on the subject’s latest diagnosis and gender. We noticed a more rapid decline among female dementia cases versus male dementia cases around 80 years of age. This suggests the nonlinear and distinct patterns of disease progression across groups within a disease. We observed distinct longitudinal trajectory patterns, which might be a possible way to monitor disease progression (further investigation of trajectory patterns is out of scope of this work).

#### EHR data

EHRs are a systematic collection of patients’ healthcare records in a digital format. EHRs are adopted in many hospitals in the US and UK.^24^ We applied the Aligned-UMAP on the MIMIC-III Critical Care Database,^17^ which consists of records of more than 40,000 patients in intensive care units (ICUs) of the Beth Israel Deaconess Medical Center between 2001 and 2012. We preprocessed the dataset following the methodology proposed by Lin et al.^25^ Figure 2E shows the lower-dimensional space on the MIMIC-III dataset from measurements recorded during the initial 72 h of entry to the ICU. We color the trajectories based on the type of critical care unit a patient stays in just before discharge from the hospital. We observe that UMAP could not recover time-related patterns; however, Aligned-UMAP segregates trajectories based on the patient’s critical care unit. This pattern reflects that it might be helpful to analyze ICU datasets stratified by their care unit and suggests that the quality of care in ICUs is highly variable.

#### COVID-19 proteomics data

Uncovering protein signatures associated with COVID-19 infection and severity can provide insights into its pathophysiology and immune dysfunction.^18^ We utilized longitudinal proteomic data on 306 COVID-19 patients.^18^ Aligned-UMAP has identified distinct trajectories for severe and nonsevere patients over 7 days (Figure 2F). We observed the participants exhibiting continued negative symptom trajectories at 7 days belonging to more severe or longer COVID-19 infection.

#### iPSC-derived neuron proteomics data

Aligned-UMAP can be incorporated as a quality control measure for longitudinal data. We applied this approach to longitudinal proteomic profiling of the differentiation of iPSC (induced pluripotent stem cell)-derived neurons cultured in different bioreactors.^20^ We could visualize distinct patterns of change for each cell line grouped by their culture environment, thereby identifying batch effects (Figure 2G). We observed that the cell lines cultured only in the 2D bioreactor are hypervariable for almost all time points (until day 28). The cell line 2D_3D (day 0–3 2D culture, day 4–28 3D culture) tends to converge around day 14, and the cell line cultured in the 3D bioreactor tends to be more homogeneous after around day 7. A tighter spread denotes a homogeneous group.

## Discussion

### Observed meaningful patterns

Our work demonstrates that Aligned-UMAP could help us discover meaningful longitudinal patterns by color coding them based on multiple known covariates. Our analysis finds that both UMAP and Aligned-UMAP help generate intuitive embeddings because of their ability to preserve the global structure. Additionally, Aligned-UMAP provides a view that highlights longitudinal structure by imposing time constraints in the embeddings, thereby controlling the stochasticity of its cross-sectional counterpart. We observe distinct trajectory patterns of the data from different modalities. Dementia and PD subtypes are delineated using clinical assessment measurements from the PPMI and ADNI studies (Figures 2A and 2B). Aligned-UMAP has also shown visually meaningful patterns on high-dimensional omics data such as proteomics (Figures 2F and 2G) or single-cell transcriptomics data (Figure 2C). Therefore, it is evident that Aligned-UMAP provides meaningful representations and is likely to be a valuable tool for researchers working on multi-variate longitudinal datasets by preserving the global and local trends along the time axis.

### Points to remember

Based on our observations from this study, this approach promises to be useful in many other biomedical datasets. These datasets can vary in terms of data missingness, time sequences, or domain-specific variations that make it challenging to tune experimental settings. So, here we discuss key points that users should keep in mind while using Aligned-UMAP.

**Figure 3:**
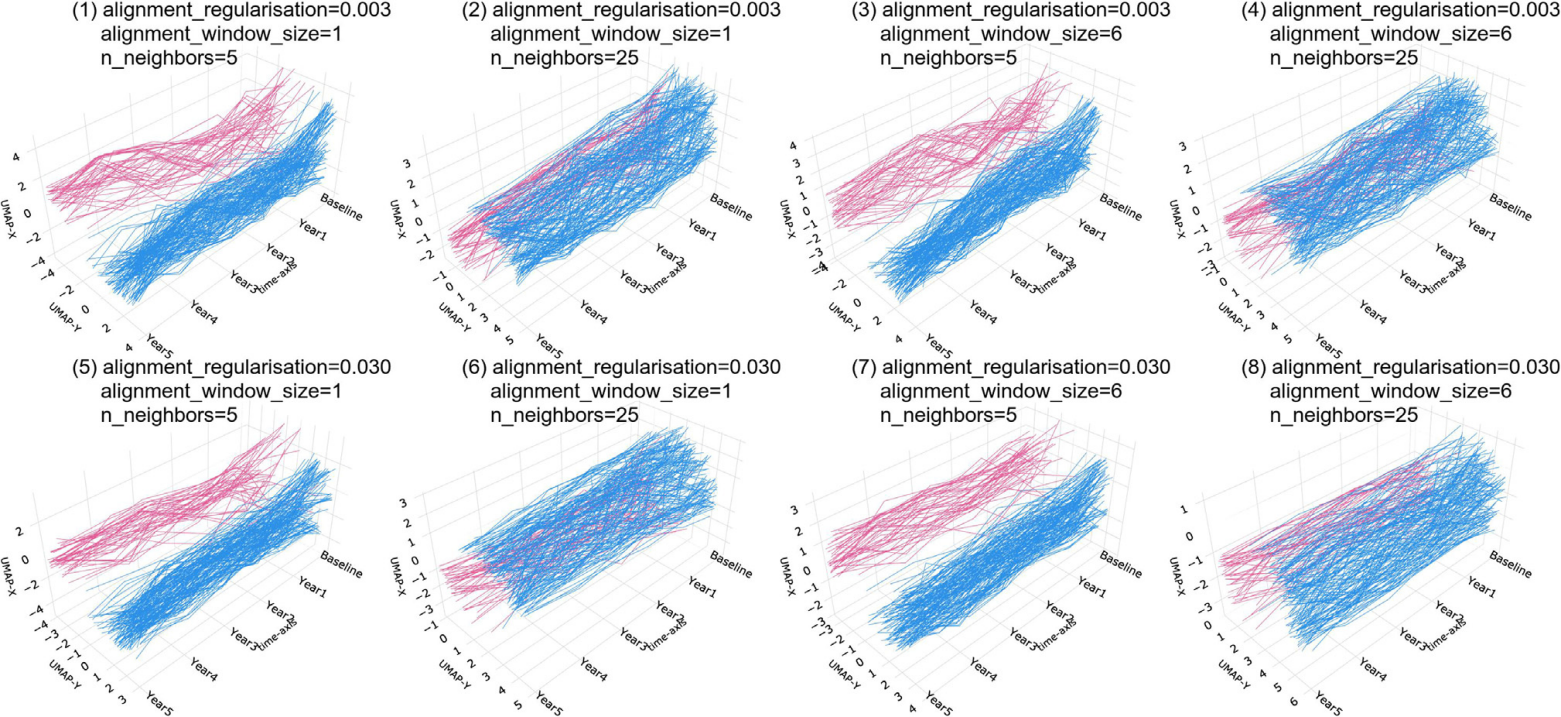
Effect of hyperparameters of Aligned-UMAP on the PPMI clinical dataset The alignment regularization is varied for [0.003, 0.03], alignment window size from [1, 6], and number of neighbors from [5, 25]. We could observe that an increase in the number of neighbors increases the size of visible clusters (1, 2). Alignment regularization and alignment window size are parameters of Aligned-UMAP that controls the volatility of trajectories. Higher values for alignment regularization will keep the related embeddings closer (1, 5), and alignment window size captures how far forward and backward across the datasets we look at when doing alignment (1, 3).
• Execution time: we analyze the execution time taken by both algorithms on multiple datasets and use their subsamples of different sizes. Further, to understand the algorithm’s scalability and parallelization, we executed it utilizing different numbers of cores (Figure 4). A multi-core setup does not seem to improve run times of Aligned-UMAP in low-data regimes, which may be attributed to intercore synchronization overheads. However, significant improvements are observed on complete lung scRNA data with 16 cores (Figure 4A). Compared with UMAP, Aligned-UMAP would require a larger dataset to have better parallelization on a multi-core machine (Figure 4B).

• Data missingness effect: the problem of missing data is prevalent in healthcare datasets and can interfere with the conclusions drawn from the data. Aligned-UMAP can handle data missingness across the longitudinal dimension by performing interpolation in low-dimensional space. Tensor decomposition-based dimension reduction approaches cannot handle any data missingness.^9^ However, none of the dimension reduction approaches are designed to handle missingness for features measured cross-sectionally.
• Aligned-UMAP parameter effect: the number of neighbors and the minimum distance are two critical parameters affecting the lower-dimensional space using the UMAP algorithm. In Aligned-UMAP, the number of parameters can increase significantly. We can vary the UMAP parameters for each step to observe different trajectories. The two other alignment parameters, namely, alignment window size and alignment regularizer, are critical in visualizing the longitudinal trend that controls the volatility along the time axis. Figure 3 shows the effect of alignment window size, alignment regularizer, and the number of neighbors on the PPMI longitudinal dataset. Our web app also demonstrates the impact of these parameters on the lower-dimensional space.

**Figure 4:**
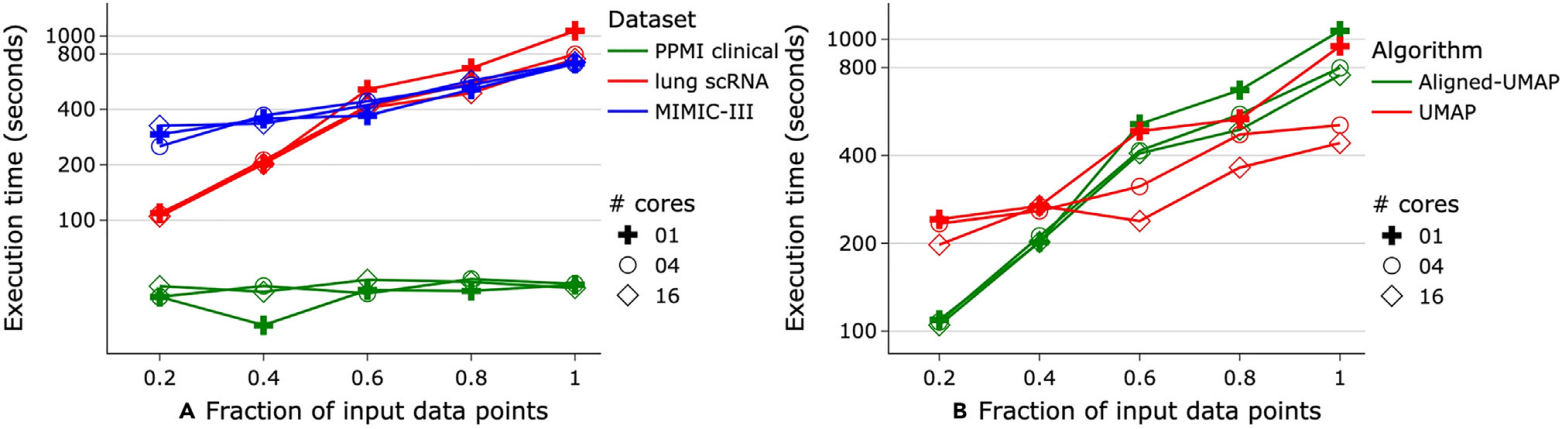
Execution time for input datasets of varying sizes (A) Comparison of Aligned-UMAP on multiple datasets. (B) Comparison of Aligned-UMAP with UMAP on whole-lung scRNA dataset. All experiments are conducted on a 128 GB RAM machine utilizing a different number of cores (marker symbol).
• Stochastic models and reproducibility: although Aligned-UMAP can handle stochasticity along the longitudinal axis, it still produces variable embeddings on different runs. Like UMAP, it uses randomness both to speed up approximation steps and to aid in solving optimization problems, thereby affecting the reproducibility of the lower-dimensional space. However, UMAP and Aligned-UMAP provide relatively stable results when applied to large amounts of data. In the future, sophisticated approaches are required to ensure reproducibility.

### Future work

The Aligned-UMAP algorithm is still in the development phase. We discuss the plausible extensions of the algorithm that might be useful in a multitude of biomedical research datasets.

• Clustering: the dimensionality reduction method is a standard preprocessing step to utilize density-based clustering methods on the high-dimensional dataset. Dynamic time warping is the most common metric to cluster time-varying patterns using K-mean clustering. It will be interesting to evaluate multiple clustering approaches on longitudinal trajectories.
• Semi-supervised/supervised: sometimes, we would like to incorporate target label information to project high-dimensional data to lower-dimensional space in dimensionality reduction. There are various reasons for supervised dimension reduction: first, to retain the internal structure of classes and have dense clusters; second, to maintain the global structure, i.e., preservation of interrelationships among the known classes; and finally, we can observe outliers or subjects that do not belong to either class using the semi-supervised learning approach. The extension of Aligned-UMAP for supervised/semi-supervised dimension reduction will be a part of future work.
• Rare events detection: the UMAP algorithm supports the detection of outliers using the local outlier factor^26^ algorithm. Identifying outliers from longitudinal trajectories generated by Aligned-UMAP will need further investigation.
• Multi-modal aspect: in the biomedical domain, monitoring disease needs data from multiple modalities such as imaging, blood biomarkers, genetics, or multi-omics.^27^^,^^28^ Current dimensionality reduction approaches are designed for datasets with single modality. The trivial way of incorporating multi-modal data is to use vectorization, but it might not be the optimal solution to discover hidden patterns in the data. Therefore, evaluating and building new dimensionality reduction approaches for multi-modal data analysis setup is required.
• Interpretability: it is important to note that because UMAP and t-SNE both necessarily warp the high-dimensional shape of the data when projecting to lower dimensions, any given axis or distance in lower dimensions still is not directly interpretable in the way of techniques such as PCA. However, PCA is highly influenced by outliers present in the data, and its inability to capture nonlinear dependencies causes a mix up among underlying clusters in lower-dimensional space.
• Data frequency: since Aligned-UMAP creates a lower-dimensional space for every location, analyzing data collected at an extremely fine scale, such as ICU or ECG spectrograms, becomes expensive.

## Experimental procedures

### Resource availability

#### Lead contact

Requests for information and resources used in this article should be addressed to Dr. Faraz Faghri (faraz@datatecnica.com).

#### Material availability

This study developed an interactive dashboard (https://alignedumap-biomedicaldata.streamlit.app/) where researchers can investigate our analysis and observe improved visualizations.

### Methods

#### Data preprocessing

All datasets went through data processing before applying the Aligned-UMAP algorithm. We follow the same methodology used in the cited publications (Table 1). Here, we list the summary of the data processing details for each of the datasets used in this work.

• PPMI clinical data^11^^,^^12^^,^^13^: these clinical data were obtained from the PPMI (http://www.ppmi-info.org/). Data went through triage for missing data, a 60 month assessment, and comprehensive phenotype collection. In the study, we included only data from participants with 60 months of follow up for PPMI. Overall, in the PPMI (n = 294 PD cases including 99 [34%] female; 154 controls including 58 [38%] female) passed the triage. We color the trajectory based on progression-based subtypes obtained from Dadu et al.^12^ We used the source code located at https://github.com/anant-dadu/PDProgressionSubtypes.
• ADNI clinical data^14^^,^^15^: clinical assessment data for Alzheimer’s disease were obtained from the ADNI database (https://adni.loni.usc.edu/). The total scores and subscores from commonly collected cognitive, functional, and longitudinal clinical data elements were aggregated to form a 78-dimension feature vector. Missing values were imputed using linear interpolation based on the past visit readings for the feature, avoiding any influence of other observations during data imputation as per Satone et al.^14^ For our analysis, we utilized the code provided at https://github.com/NIH-CARD/ADProgressionSubtypes.
• PPMI-ADNI T1 MRI^13^^,^^15^^,^^16^: in this dataset, we used derived features that include regional brain volumes, cortical thickness, and area as T1 MRI imaging features. We used ANTsPyT1w available at https://github.com/stnava/ANTsPyT1w to preprocess the images.
• MIMIC-III^17^: we utilized the data processing code available at https://github.com/Jeffreylin0925/MIMIC-III_ICU_Readmission_Analysis to generate features from EHRs. We used three categories of features in this work, namely chart events, ICD-9 embeddings, and demographic information of the patients.^25^

We download the preprocessed version for the other three datasets using the link provided in the relevant publications, longitudinal proteomic COVID-19 from Filbin et al.,^18^ longitudinal whole-lung scRNA from Strunz et al.,^19^ and iPSC derived neurons from Reilly et al.^20^ On all these datasets, we applied min-max normalization to numerical features to preserve the longitudinal relationships among the original data and ensure a zero-to-one range. Additionally, we outlined the specifics of data preparation in the readme file of our publicly accessible GitHub repository (https://github.com/NIH-CARD/AlignedUMAP-BiomedicalData#step1-prepare-data).

#### Statistical and machine learning analyses

After preparing the data, we perform unsupervised machine learning using the Aligned-UMAP algorithm. We hypothesized that this approach could identify the clusters with distinct trajectories over time. Since this work is an entirely unsupervised analysis, we visualize 3D trajectory plots, color coded based on metadata, to evaluate the algorithm’s performance. We performed extensive hyperparameter tuning with different sets of values for Aligned-UMAP parameters (distance metric, alignment regularization, alignment window size, number of neighbors, minimum distance). For additional information, please see section 2 of the readme file available in our GitHub repository at https://github.com/NIH-CARD/AlignedUMAP-BiomedicalData#step2-setup-configuration-and-data-paths. Finally, we analyze the time taken by Aligned-UMAP on all our datasets to provide the estimate of execution time to the users (Figure 4).

## Data Availability

The data used in this study was access controlled from the PPMI (http://www.ppmi-info.org/) and the ADNI (https://adni.loni.usc.edu) and require individual sign up to access the data. EHRs from MIMIC-III Critical Care Database were downloaded from PhysioNet: https://physionet.org/content/mimiciii-demo/1.4. Bulk and scRNA-seq data from mice whole lung are available via the Gene Expression Omnibus with the accession code GEO: GSE141259. COVID-19 longitudinal proteomic data have been downloaded from Mendeley Data: https://doi.org/10.17632/nf853r8xsj. Additionally, we have developed an interactive website (https://alignedumap-biomedicaldata.streamlit.app/) where researchers can investigate components of the predictive model and can investigate feature effects on a sample and cohort level. All other data reported in this descriptor will be shared by the lead contact upon request. Any additional information required to reanalyze the data reported in this descriptor is available from the lead contact upon request. To facilitate replication and expansion of our work, we have made the notebook publicly available on GitHub at https://github.com/NIH-CARD/AlignedUMAP-BiomedicalData. It includes all code, figures, models, and supplements for this study. The code is part of the supplemental information; it includes the rendered Jupyter notebook with full step-by-step data preprocessing, statistical, and machine learning analyses. All original code has been deposited at Zenodo under Zenodo: https://doi.org/10.5281/zenodo.7562874 and is publicly available as of the date of publication (Zenodo: https://doi.org/10.5281/zenodo.7562874).
